# Hairy Root Cultures as a Source of Phenolic Antioxidants: Simple Phenolics, Phenolic Acids, Phenylethanoids, and Hydroxycinnamates

**DOI:** 10.3390/ijms24086920

**Published:** 2023-04-07

**Authors:** Janusz Malarz, Yulia V. Yudina, Anna Stojakowska

**Affiliations:** 1 Maj Institute of Pharmacology, Polish Academy of Sciences, Smętna Street 12, 31-343 Kraków, Poland; 2 Educational and Scientific Medical Institute, National Technical University “Kharkiv Polytechnic Institute”, Kyrpychova Street 2, 61002 Kharkiv, Ukraine

**Keywords:** *Agrobacterium rhizogenes*, caffeoylquinic acid, chlorogenic acid, chicoric acid, phenolic acid, salvianolic acid, *Rhizobium rhizogenes*, rosmarinic acid, salidroside, verbascoside

## Abstract

Plant-derived antioxidants are intrinsic components of human diet and factors implicated in tolerance mechanisms against environmental stresses in both plants and humans. They are being used as food preservatives and additives or ingredients of cosmetics. For nearly forty years, *Rhizobium rhizogenes*-transformed roots (hairy roots) have been studied in respect to their usability as producers of plant specialized metabolites of different, primarily medical applications. Moreover, the hairy root cultures have proven their value as a tool in crop plant improvement and in plant secondary metabolism investigations. Though cultivated plants remain a major source of plant polyphenolics of economic importance, the decline in biodiversity caused by climate changes and overexploitation of natural resources may increase the interest in hairy roots as a productive and renewable source of biologically active compounds. The present review examines hairy roots as efficient producers of simple phenolics, phenylethanoids, and hydroxycinnamates of plant origin and summarizes efforts to maximize the product yield. Attempts to use *Rhizobium rhizogenes*-mediated genetic transformation for inducing enhanced production of the plant phenolics/polyphenolics in crop plants are also mentioned.

## 1. Introduction

Hairy roots (HRs) emerge as a result of transfer and permanent incorporation of a foreign genetic material, delivered by the fragment (T-DNA) of the bacterial Ri plasmid into the plant genomic DNA. *Rhizobacterium rhizogenes* (formerly *Agrobacterium rhizogenes*), a plant growth-promoting Gram-negative bacterium that is a carrier of the Ri plasmid, has been known since the 1930s of the twentieth century as the etiological agent of the “hairy root disease”. Attempts to apply the HRs for the exploration of plant secondary metabolite biosynthesis started in the middle of 1980s [1,2]. Since then, HRs of numerous plant species have been described, and their biosynthetic potential has been thoroughly studied [3,4,5]. The advantages of the HR cultures are as follows: the rapid growth in nutrient media with reduced concentration of macronutrients and without plant growth regulators, the biosynthetic potential similar to that of the parent plant, and the ability to grow and synthesize natural products in the dark. The major drawbacks are unsatisfactory upscaling results (the need for large-volume bioreactors) and unsatisfactory costs/profit ratio. Along with the studies of plant metabolism, HRs have been found to have use in plant breeding, genetic engineering and research on gene functions, phytoremediation, and biotransformation [6]. The most promising area of their industrial application seems to be the production of recombinant proteins and production of specialized metabolites [3]. However, the process should be technically and economically feasible. Plant secondary metabolites of medical applications have dominated the research on natural products biosynthesized with HRs for decades. Recently, production of food ingredients and food by plant cell and tissue cultures has gained growing interest. The current regulations, however, do not allow the use of genetically transformed cultures for the production of commercial food products and ingredients [7].

Mechanism of T-DNA transfer from *R. rhizogenes* into the nucleus of a plant cell and subsequent HR formation, the bacterial *rol* and *aux* genes mechanisms of action, the HRs’ cultivation methods including their cultivation in bioreactors of different scale and design, methods applied to induce HRs’ growth and to enhance biosynthesis of secondary metabolites have been the subject of numerous review papers. Majority of the reviews presented lists of plant species transformed with *R. rhizogenes* together with the principal secondary metabolites synthesized by the obtained HRs. However, quantitative data that are of key importance for the assessment of HR prospects for application are usually missing.

Phenolics/polyphenolics and some terpenoids (e.g., carotenoids) are well-known plant secondary (specialized) metabolites of antioxidative and chemopreventive activity [8,9,10,11] that help combat lifestyle diseases [12,13,14,15]. Although the compounds are constituents of fruits and vegetables that are recommended components of human diet, their bioavailability often remains low and is strongly affected by food processing, food matrix, and gut microbiota [16,17,18]. Therefore, isolated plant polyphenols may be used for food supplementation and enrichment. Due to their antioxidative and antimicrobial properties, phenolics of plant origin may find application as food preservatives [19,20,21]. Antioxidative, antimelanogenic, and UV-B protecting activities of plant phenolic compounds also make them good candidates for antiaging skin care ingredients [22,23,24]. Apart from the application in the food and cosmetic industry, the compounds have garnered interest as potential natural herbicides and fungicides [25].

Although technological and economical limitations still hamper industrial use of HRs as a source of phytochemicals, a growing demand for high added-value molecules from plants and requirements for biodiversity protection may speed up the implementation of processes based on large-scale HR cultures. The objective of this review is to summarize the achievements in phenolic/polyphenolic production by HRs in order to show the current possibilities and limitations of the technique.

In our previous paper [26], we summarized the attempts to produce flavonoids, stilbenoids, and hydrolysable tannins in HRs. However, plant polyphenolics are a much larger group of natural products and encompass further subclasses of plant metabolites. The present review is based on the experimental results concerning the production of simple phenolics, hydroxycinnamates, and phenylethanoids in HRs that were published before February 2023 in journals covered by two databases: Web of Science and Scopus. The search terms used were the following: “hairy roots”, “*Agrobacterium rhizogenes*”, or “*Rhizobium rhizogenes*” in conjunction with “phenolics”, “phenolic acids”, “phenols”, “polyphenols”, “hydroxybenzoic”, “hydroxycinnamic”, “chlorogenic”, “caffeic”, “ferulic”, “coumaric”, “phenylethanoid”, “phenylpropanoid” “tyrosol”, “salidroside”, “acteoside”, “verbascoside”, “echinacoside”, “martynoside”, “chicoric acid”, “cichoric acid”, “caftaric acid”, “caffeoylquinic”, “rosmarinic acid”, “lithospermic acid”, and “salvianolic acid”. No date range was set in order to include more data. As a result, over two hundred research papers that contained quantitative data were analyzed in detail.

## 2. Phenolic and Polyphenolic Antioxidants in HRs

Phenolic compounds are present in all vascular plants. They occur in plant cells either in soluble form or bounded to cell walls. The natural products originate from the shikimate pathway that, via chorismate and prephenate, leads to aromatic amino acids tyrosine and phenylalanine. The latter compound is deaminated by phenylalanine ammonia lyase (PAL) to *trans*-cinnamic acid, and the reaction is the first committed step in the phenylpropanoid pathway that is involved in the biosynthesis of polyphenols [27,28].

### 2.1. Simple Phenolics and Phenolic Acids

**Figure 1:**
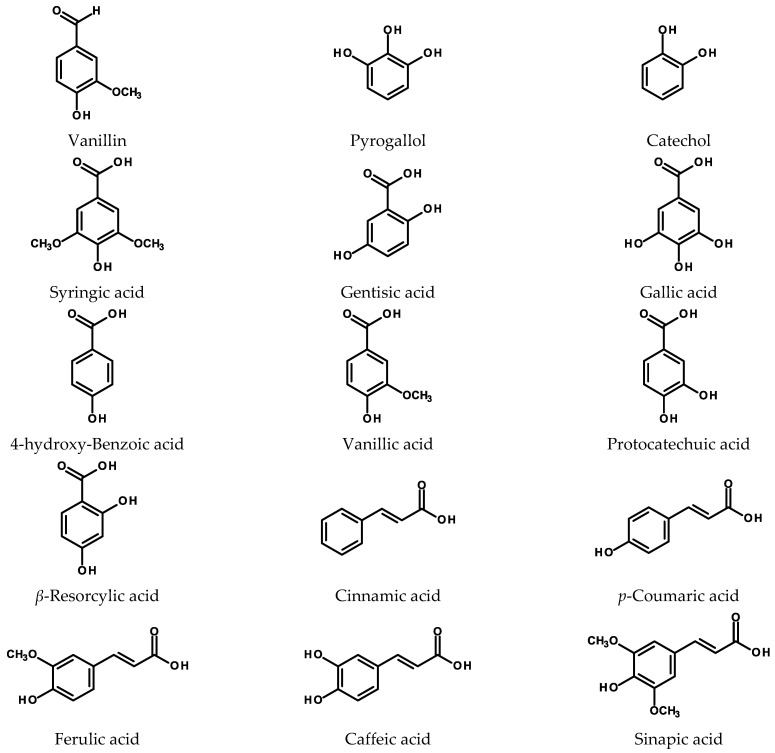
Chemical structures of selected simple phenolics and phenolic acids.

The most common types of the simple phenolics occurring in plants (for structures see Figure 1) are the hydroxybenzoic (C_6_-C_1_), phenylacetic (C_6_-C_2_), and hydroxycinnamic (C_6_-C_3_) acid derivatives as well as the phenolic alcohols (C_6_, C_6_-C_1_, C_6_-C_2_, and C_6_-C_3_). Some of them are the building blocks for the more complex molecules (polyphenolics), such as coumarins, lignans, depsides, and conjugates of phenols with sugars and organic acids. Hydroxybenzoic and hydroxycinnamic acid derivatives are common constituents of foods and beverages that, in some instances, are easily available from postproduction waste or side products in the food industry [29,30]. Their presence in the HRs is usually recorded together with polyphenolics of more complex structure.

Maximum contents of soluble phenolics in the HRs were shown to not usually exceed 0.1% calculated on a dry weight (DW) basis. Cell wall-bound phenolics were present in greater amounts. For example, the cell wall-bounded and soluble form of *p*-hydroxybenzoic acid (*p*-HBA, 4-HBA) constituted up to 0.44% and up to 0.04% DW, respectively, in HRs of *Daucus carota* L. (Apiaceae) elicited with chitosan [31]. The highest titers of *p*-HBA derivatives in the soluble phenolic fraction have been found in the *Beta vulgaris* L. (Amaranthaceae) and the *Datura stramonium* L. (Solanaceae) HRs expressing 4-hydroxycinnamoyl-CoA hydratase/lyase (HCHL) of bacterial origin [32,33]. The enzyme hydrates 4-hydroxycinnamoyl-CoA thioesters and then catalyzes a retro-aldol cleavage to produce the corresponding 4-hydroxybenzaldehydes and acetyl-CoA. The HRs with heterologous expression of *HLCL* demonstrate a diminished availability of feruloyl-CoA, an increased bounding of *p*-HBA to the cell wall, and a high content of the *p*-HBA derivatives (glucoside and glucose ester of *p*-HBA, *p*-hydroxybenzyl alcohol glucoside) in the soluble phenolic fraction. The most productive line of the *HLCL*-transgenic *D. stramonium* HRs accumulates up to 0.81% of the *p*-HBA-related compounds, calculated on the fresh weight (FW) basis [32]. One of the *HLCL*-transgenic *B. vulgaris* root clones was shown to accumulate nearly 14% DW of *p*-HBA glucose ester [32] while 4-Hydroxybenzoic acid-*β*-D-glucoside has been found to be a major compound in the *HLCL*-transgenic *D. stramonium* HRs.

The HRs demonstrate the potential to convert exogenously applied phenolic compounds into their glucosides. In one study, *R. rhizogenes* transformed roots of *Lobelia sessilifolia* Lamb. (Campanulaceae) glucosylated protocatechuic acid to its 3-O-*β*-D-glucopyranoside [34]. The HRs of *Polygonum multiflorum* Thunb. (Polygonaceae) glucosylate 4-hydroxybenzene derivatives to the corresponding 4-*β*-D-glucopyranosides [35]. The HR culture of *Panax ginseng* C.A. Mey. (Araliaceae) performed a biotransformation of two HBA isomers (*m*-HBA and *p*-HBA) into their corresponding *β*-D-glucopyranosides and *β*-D-glucopyranosyl esters whereas the products of *o*-HBA biotransformation were not detected [36].

Sircar et al. [37] found that the HRs of carrot (*D. carota*) that accumulated *p*-HBA may be a good experimental system to study the biosynthesis of this compound. The carrot HRs elicited with methyl jasmonate (MeJa, 100 μM) demonstrated higher content of cell wall-bounded *p*-HBA (0.42% DW) than did the control roots (0.18% DW). A detailed analysis of the enzymatic activities in cell-free extracts from the HRs elicited with MeJa revealed that the elevated activities of PAL and hydroxybenzoate dehydrogenase (HBD), that converts *p*-hydroxybenzaldehyde to *p*-HBA, preceded enhanced accumulation of *p*-HBA in the elicited roots. Moreover, the enhanced depletion of *p*-coumaric acid with subsequent formation of *p*-hydroxybenzaldehyde, catalyzed by the cell-free extract from the elicited roots, indicated the activity of *p*-hydroxybenzaldehyde synthase (HBS), an enzyme catalyzing phenylpropanoid side-chain cleavage of *p*-coumaric acid [38]. The inhibition of PAL and cinnamate-4-hydroxylase (C4H) in the *D. carota* HRs elicited with chitosan (200 mg/L) abolished the enhanced accumulation of *p*-HBA and *p*-coumaric acid by the elicited roots. The inhibition of 4-coumarate:CoA ligase (4CL) did not affect *p*-HBA or *p*-coumarate biosynthesis. The maximum content of the soluble (0.05% DW) and cell wall-bound (0.45% DW) *p*-HBA in the roots was achieved upon simultaneous elicitation with chitosan and precursor feeding at day 15 of the culture cycle [31]. The MeJa-elicited HRs of carrot, treated with inhibitors of alternative oxidase (AOX), i.e., salicylhydroxamic acid (SHAM) and propyl gallate (PG), demonstrated a reduced accumulation of phenolic acids, flavonoids and lignin, although transcript levels of *PAL*, *DcAOX2a*, and *DcAOX1a* significantly increased after the MeJa treatment. Thus, biosynthesis of phenylpropanoid derivatives in the MeJa-treated HRs was found to be positively associated with AOX activity [39]. The exposure of the *D. carota* HRs to continuous light (250 μmol m^−2^ s^−1^) induced a photooxidative stress response in the root tissue. The roots turned green and exhibited an enhanced superoxide dismutase (SOD) activity. The transfer of the carrot HRs from dark to light conditions changed the metabolism of the roots. HBD, shikimate dehydrogenase (SKDH), and PAL activities were suppressed, whereas activities of the 1-deoxy-_D_-xylulose 5-phosphate reductoisomerase (DXR) and pyruvate kinase (PK) were enhanced in the green HRs as compared to the normal ones. Gene expression analysis for DXR and PAL showed trends similar to those for the respective enzyme activities. The green roots accumulated 50% less of the cell wall-bound *p*-HBA than did the normal HRs cultured in dark but the production of volatile mono- and sesquiterpenoids was enhanced in the light-exposed roots [40,41].

Gentisic acid (0.17 mg/g DW) and ferulic acid (0.33 mg/g DW) were found to be the most abundant phenolic acids in the HRs of *Brassica rapa* L. ssp. rapa (Brassicaceae). The roots of in vitro grown *B. rapa* plantlets contained 0.18 mg/g DW and 0.27 mg/g DW of the acids, respectively [42]. Elicitation of the HRs with silver nanoparticles (100 mg/L; 48 h) caused an increase in the phenolic acid contents up to 0.20 mg/g DW (gentisic acid) and 0.39 mg/g DW (ferulic acid). The elicited HRs demonstrated an enhanced antimicrobial and radical scavenging activity and an enhanced expression of *PAL* [43]. Gentisic and ferulic acid were also major phenolic acids present in the HRs of *B. rapa* ssp. *pekinensis*. Their contents reached 0.68 mg/g DW and 0.56 mg/g DW, respectively, upon elicitation with copper oxide nanoparticles (100 mg/L) [44].

The HRs obtained by the inoculation of *Momordica charantia* L. (bitter melon, Cucurbitaceae) explants with *R. rhizogenes* KCTC 2703-synthesized gentisic (5.53 mg/g DW) and salicylic (SA; 2.52 mg/g DW) acid as major components of phenolic acid fraction. The spine gourd (*Momordica dioica* Roxb. ex Willd.) HRs produced much lower amounts of the phenolics (0.54 and 0.49 mg/g DW, respectively) [45,46]. Gentisic acid and SA were also the predominant hydroxybenzoic acids in the gherkin (*Cucumis anguria* L.) HRs (0.76 mg/g DW and 1.00 mg/g DW, respectively) and nontransformed roots of the plant (0.55 and 0.74 mg/g DW) [47].

The *P. multiflorum* HRs developed from leaf explants infected with *R. rhizogenes* KCTC 2703, except for hydroxycinnamic and hydroxybenzoic acids (gallic acid: 0.56 mg/g DW), were shown to accumulate pyrogallol (1.37 mg/g DW). The culture produced up to 10.95 g of dry biomass per 1 L of a nutrient medium in 20 days [48]. In the HRs of *P. multiflorum* obtained after the inoculation with *R. rhizogenes* KCCM 11879, flavonoids dominated the phenolic compound fraction [49]. The roots contained *p*-HBA and ferulic acid (3.46 mg/g and 2.20 mg/g DW, respectively) as well as several other phenolic acids in minor quantities. Five days after the addition of MeJa (50 μM) to the culture medium, a 5.48-fold increase in the *p*-HBA content and a 4.3-fold increase in the SA content was observed. Pyrogallol has not been mentioned as a metabolite of the culture.

The current status of research on the buckwheat (*Fagopyrum esculentum* Moench and *Fagopyrum tataricum* (L.) Gaertn.; Polygonaceae) HRs has been recently summarized by Tomasiak et al. [50]. The transformed buckwheat root cultures accumulate a number of phenolic acids, of which caffeic and ferulic acids were reported to be the most abundant ones (up to 1.0 mg/g DW).

The HRs of two *Ficus carica* L. (Moraceae) cultivars, cv. Sabz and cv. Siah, were obtained by the inoculation of leaf explants with *R. rhizogenes* line ATCC 15834 and A7, respectively. Upon the elicitation with MeJa (100 μM), the HRs of cv. Siah produced up to 0.71 mg/g DW of gallic acid and up to 6.5 mg/g of chlorogenic acid (5-CQA; IUPAC numbering, see Section 2.3.2). The HRs of cv. Sabz, after the elicitation with MeJa (200 μM), accumulated gallic (0.19 mg/g DW), caffeic (0.28 mg/g DW), chlorogenic (1.63 mg/g DW), and coumaric (0.23 mg/g DW) acids [51]. The same root culture elicited with the *Piriformospora indica* fungal cell extract or culture filtrate, at a concentration range of 2–6% (*v*/*v*), demonstrated an enhanced production of phenolic acids (up to 7.49 mg/g of gallic acid, up to 0.83 mg/g of caffeic acid, up to 1.58 mg/g of 5-CQA, and up to 0.94 mg/g of coumaric acid, as calculated on a DW basis). The most effective phenolic biosynthesis stimulant was the *P. indica* culture filtrate, at 2% (*v*/*v*) concentration, that was a potent inducer of *PAL* expression. The lowest concentration of both the fungal cell extract and the fungal culture filtrate strongly promoted the growth of the HRs [52].

*Aster scaber* Thunb. (Asteraceae) explants infected with *R. rhizogenes* KCTC 2703 were found to produce HRs that accumulated simple phenolics with SA (0.32 mg/g DW) as the major compound. The elicitation with MeJa (100 μM, 96 h treatment) enhanced the accumulation of SA up to 0.44 mg/g DW [53]. In the HRs of *Ligularia fischeri* Turcz. f. *spiciformis* (Nakai) (Asteraceae), SA (0.30 mg/g) and caffeic acid (0.18 mg/g DW) dominated the hydroxybenzoic and hydroxycinnamic acids fractions, respectively [54]. The main phenolic acid found in the HRs of *Sphagneticola calendulacea* (L.) Pruski (Asteraceae) was ferulic acid (0.21 mg/g DW). Feeding the roots with phenylalanine (0.5 mM added on day 10 of the culture), increased the ferulic acid content up to 1.19 mg/g DW [55].

Numerous wild-type and genetically modified HR cultures of Lamiaceae plants have been established for the studies on secondary metabolism. Expression of *Arabidopsis* PAP1 (Production of Anthocyanin Pigment 1 transcription factor from the MYB family) in the HRs of *Leonurus sibiricus* L. led to the enhanced production of hydroxycinnamic acids. Contents of the analyzed compounds were correlated with the relative expression levels of *AtPAP1*. The most productive of the *AtPAP1*-carrying HR clones accumulated up to 19.39 mg/g DW of chlorogenic acid, 11.38 mg/g DW of caffeic acid, and 1.17 mg/g DW of ferulic acid [56]. *Mentha spicata* L. roos transformed with *R. rhizogenes* A13, upon the elicitation with MeJa (100 μM), demonstrated an enhanced expression of *PAL*, *4CL*, and *C4H* with a subsequent increase in cinnamic acid content. The contents of caffeic and chlorogenic acids were significantly higher than those in the control roots 3–6 h and 72–96 h after the addition of MeJa, respectively [57]. The HRs of *Leonotis nepetifolia* (L.) R. Br., obtained by infecting of the aseptically grown seedlings with *R. rhizogenes* strain A4, contained substantial amounts of *p*-coumaric acid (4.91 mg/g DW) and *m*-coumaric acid (2.05 mg/g DW) [58].

Kochan et al. [59] compared quantities of phenolic acids accumulated by the roots of three-year-old field-grown *Panax quinquefolius* L. (Araliaceae) plants with those in the wild-type HRs of the plant cultivated in shake flasks and a nutrient sprinkle bioreactor. In the bioreactor-cultivated HRs, a significant drop in *p*-HBA, syringic, and gentisic acid contents was observed. The HRs tended to accumulate higher contents of 5-CQA than did the roots of field-grown plants.

Ferulic acid was found to dominate a cell wall-bound fraction of phenolics in the HRs of tomato (*Lycopersocum esculentum* Mill. cv. Arka Suarabh; Solanaceae). Upon the elicitation with chitosan (1 mL of 1% solution in 60 mL of a nutrient medium) or fungal preparations from *Fusarium oxysporum* f. sp. *lycopersici* and *Trichoderma viride*, the content of ferulic acid in HRs rose from 0.3 (control HRs) to 1.0 mg/g DW (elicited HRs, 24 h after the induction) [60].

β-Cryptogein is a basic polypeptide of elicitin family, produced by the plant pathogenic fungus *Phytophthora cryptogea*. The polypeptide triggers a defense response in plants. In one study, leaves from axenic *Nicotiana tabacum* L. (Solanaceae) plants were infected with *R. rhizogenes* strain LBA 9402 carrying either the empty vector pBIN19 or pBIN19 harboring the synthetic β-cryptogein gene under the control of a CaMV promoter. The Ri-cryptogein-transformed plants were characterized by a diminished production of H_2_O_2_ and O_2_^-^ radicals, diminished lipid peroxidation, and suppressed levels of transcripts for the isoprenoid biosynthetic pathway enzymes. Simultaneously, the plants exhibited enhanced activities of antioxidative enzymes (peroxidase, ascorbate peroxidase, catalase, SOD), higher lignin content, and higher cell wall-bound phenolics content [61].

To obtain the HRs of *Pelargonium sidoides* DC. (Geraniaceae), a medicinal plant that produces coumarins of unique structure, *R. tumefaciens* C58C1 carrying the pRiA4 plasmide was applied. The developed HRs were cultivated in the liquid media containing three different concentrations of MeJa (25, 50, and 100 μM). The elicitor, at the applied concentration range, enhanced the accumulation of hydroxybenzoic acids: gallic acid (maximum content: 2.43 mg/g DW; 50 μM MeJa) and vanillic acid (maximum content: 1.66 mg/g DW; 25 μM MeJa) [62].

The HR cultures that accumulated high contents of simple phenolics and phenolic acids are shown in Table 1.

### 2.2. Phenylethanoids

**Figure 2:**
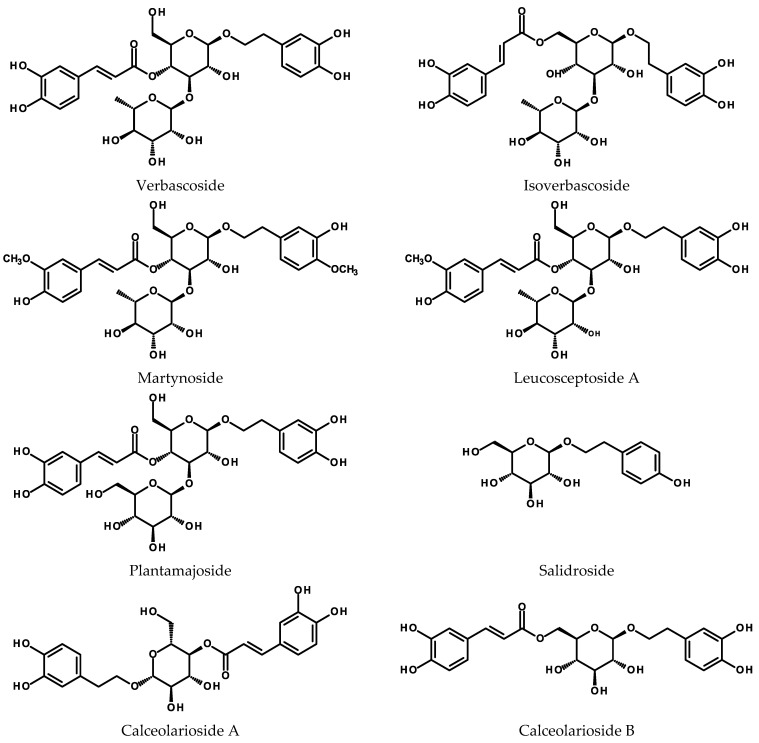
Chemical structures of selected phenylethanoids.

Studies on the phenylethanoid production in the HRs mostly discuss verbascoside biosynthesis in the roots of plant species included into the Lamiales order or salidroside biosynthesis in *Rhodiola* spp. (Crassulaceae). Both compounds (for structures see Figure 2) demonstrate numerous activities of potential application in medicine. Verbascoside (synonyms: acteoside, kusaginin, orobanchin) protects human keratinocytes against the UV radiation, scavenges free radicals, and acts as an antioxidant and chemopreventive agent. The phenylethanoid glycoside, due to its interaction with estrogenic receptors, may exert anticancer and antimetastatic effects. Moreover, it has demonstrated antiproliferative activity against some cancer cell lines in vitro and may induce differentiation or apoptosis in some types of cancer cells. Anti-inflammatory and antimicrobial activities of the compound are also worth mentioning [63,64]. Salidroside, a major constituent of rosavin complex present in *Rhodiola rosea* L. roots, exerts antioxidant, cytoprotective, and antitumor activity [64,65]. A review on the pharmacological activity of salidroside with the focus on CNS functions has been published recently by Jin et al. [66]. Another phenylethanoid glycoside, echinacoside, shares similar pharmacological activity (neuro- and hepatoprotective, anticancer, anti-inflammatory) [64]. Its biosynthesis in HRs of *Echinacea* spp. (Asteraceae) is discussed in the Section 2.3.1.

In one study, the HR culture of *Scutellaria baicalensis* Georgi (Lamiaceae) was used to synthesize five known phenylethanoid glycosides: martynoside, leucosceptoside A, verbascoside, 2-(3-hydroxy-4-methoxyphenethyl) 1-*O*-*α*-L-rhamnopyranosyl-(1 -> 3)-*β*-D-(4-*O*-feruloyl) -glucopyranoside, and 4-hydroxy-*β*-phenethyl-*β*-D-glucopyranoside [67]. Acteoside (verbascoside) was the most abundant phenolic compound found in two clones of *S. baicalensis* HRs cultivated in the dark in a modified MS (Murashige and Skoog) [68] medium with reduced (50%) concentration of KNO_3_, NH_4_NO_3_, and CaCl_2_ and doubled concentrations of KH_2_PO_4_ and MgSO_4_. The phenylethanoid glycoside content in the HRs reached 3% DW. The compound was absent from roots of field-grown plants but was detected in leaves and roots of in-vitro-grown plantlets [69]. The HRs of *S. lateriflora* L., established by inoculation of leaf explants with *R. rhizogenes* strain A4, accumulated 18.5 mg/g DW of acteoside. The elicitation of the culture with yeast extract (YE; 50 and 200 μg/mL), significantly increased acteoside content after 7 and 14 days. An addition of the bacterial lysates (*R. rhizogenes*, *Pseudomonas syringae*, *Pseudomonas carotovorum*, *Enterobacter sakazakii*, *Klebsiella pneumoniae*) at two doses (7.5 and 15 mL/L) did not stimulate the biosynthesis of acteoside. On the contrary, in the HRs treated with *Pseudomonas carotovorum* lysate on the 26th day of culture, after the following 14 days, acteoside was not detected [70]. Marsh et al. [71] examined the effects of light, elicitation with MeJa, and the addition of β-cyclodextrin on the growth and production of phenolic compounds in the HRs of *S. lateriflora* obtained from the stem explants infected with *R. rhizogenes* ATCC 15834. The maximum content of verbascoside (c. 17 mg/g DW) was detected in the roots grown in the dark and treated (for 24 h) either with 15 mM of β-cyclodextrin or with 100 μM of MeJa.

In one study, the HRs of *Paulownia tomentosa* Steud. (Bignoniaceae) were obtained from the axenic stem explants infected with *R. rhizogenes* LBA 9402. The most productive root clone, cultivated in ½ B5 medium [72], accumulated up to 94.9 mg/g DW (1.21 g/L) of verbascoside [73]. The *Catalpa ovata* G. Don. (Bignoniaceae) HRs cultivated in similar conditions produced up to 41.2 mg/g DW of verbascoside and up to 5.8 mg/g DW of martynoside [74].

In another study, two types of root culture were initiated from axenic seedlings of *Plantago lanceolata* L. (Plantaginaceae): the HRs transformed with *R. rhizogenes* LBA 9402 and the untransformed roots. Both cultures produced similar yields of verbascoside (6–12 mg/g DW) and plantamoside (plantamajoside; 30–80 mg/g DW). Plantamoside content in the HRs matched that found in the roots of the whole plant. Cinnamic acid feeding had no effect on the phenylethanoid glycoside content. Instead, cinnamic acid glucose ester accumulated in the roots [75].

In other research, seedlings of *Gmelina arborea* Roxb. (Verbenaceae) were infected with a wild-type *R. rhizogenes* strain ATCC 15834 to initiate the HR culture. Verbascoside content in the developed HRs reached 0.91 mg/g DW versus 8.4 mg/g DW in the root of the mature tree [76].

*Harpagophytum procumbens* DC. (devil’s claw; Pedaliaceae), an African plant traditionally used to treat a variety of pain and inflammatory conditions, is known to produce iridoids and phenylethanoids. In one study, the HRs of the plant were cultivated either in flasks with shaking or in a bubble column bioreactor. The roots from the bioreactor contained more iridoids than did the roots cultivated in flasks. The phenylethanoid content was not analyzed [77]. The contents of four phenyletanoid glycosides (verbascoside, martynoside, *β*-OH-verbascoside, and leucosceptoside A) were compared for the roots grown in flasks and in a stirred tank bioreactor. Both cultures accumulated verbascoside (c. 1.1 mg/g DW) as a major phenyloethanoid. Roots grown in the bioreactor contained more leucosceptoside A but less *β*-OH-verbascoside and martynoside than did the roots from flasks [78]. Two wild-type *R. rhizogenes* strains, A4 and ATCC 15834, were used to obtain the *H. procumbens* HRs by Grąbkowska et al. [79]. Verbascoside content differed among the studied HR clones (5.34–8.12 mg/g DW) and was lower than that in the roots of the intact plant (c. 10 mg/g DW). In contrast, some of the HR clones accumulated more isoverbascoside (up to 16 mg/g DW) than did the plant roots. According to Gyurkovska et al. [80], cell suspension cultures of *H. procumbens* were a better source of phenylethanoids and demonstrated better anti-inflammatory activity than did the HRs of the plant. The maximum content of verbascoside in the examined HRs reached 0.94 mg/g DW. A phenylethanoid-containing fraction from the HRs inhibited both acetylcholinesterase (AChE) and butyrylcholinesterase (BChE) at a concentration range 100–200 μg/mL, while a similar fraction from the cell suspension acted selectively against BChE. It is worth noting that the isolated phenyletanoids exerted a moderate anti-inflammatory effect [80,81].

Piątczak et al. [82] investigated the effects of the elicitation with MeJa and SA on phenyletanoide content in the HRs of *Rehmannia glutinosa* Libosch. (Orobanchaceae) transformed with *R. rhizogenes* strain A4. The 23-day-old HR cultures were treated either with MeJa or with SA (50–200 μM). A combination of the two elicitors was also tested. The highest verbascoside content (60.07 mg/g DW) was achieved with MeJa treatment (200 μM, 72 h). The elicitation with SA was used as a tool to detect genes and enzymes engaged in verbascoside biosynthesis in the HRs of *R. glutinosa* transformed with *R. rhizogenes* strain ACCC10060 [83]. The roots were treated with four elicitors (SA, MeJa, AgNO_3_, and putrescine), and SA was ultimately found to be the most effective in inducing verbascoside biosynthesis. The maximum yield of verbascoside (11.66 mg/g DW or 53.87 mg/L) was achieved by 12–24 h treatment with 25 μM/L of SA. The functional analysis of differentially expressed genes and the analysis of expression levels of the genes putatively involved in verbascoside biosynthesis (*PAL*; *C4H*; coumaroylquinate (coumaroylshikimate) 3′-monooxygenase, *C3H*; *4CL*; tyrosine/DOPA decarboxylase, *TyDC*; polyphenol oxidase, *PPO*; copper-containing amine oxidase, *CuAO*; alcohol dehydrogenase, *ALDH*; UDP-glucose glucosyltransferase, *UGT*; shikimate *O*-hydroxycinnamoyltransferase, *HCT*) were performed to elucidate the biosynthetic pathway. The HR culture of *R. elata* N.E. Brown ex Prain synthesized iridoid glycosides and phenylethanoids. The most productive of the analyzed root clones accumulated 17.35 mg/g DW of verbascoside and c. 2 mg/g DW of isoverbascoside [84].

Plants from the genus *Verbascum* (Scrophulariaceae) have long been used in traditional medicine as remedies for respiratory tract infections and disorders. Iridoid glycosides, saponins, flavonoids, and phenylethanoids are regarded as the active constituents of the plants [85]. The HRs of *V. xanthophoeniceum* Griseb. were obtained to study their capability to produce active compounds. Attempts to achieve root proliferation with three different *R. rhizogenes* strains (TR 105, ATCC 15834 and LBA 9402) by direct infection and cocultivation method failed. Finally, the HRs were obtained with the ATCC 15834 strain with sonication-assisted transformation. In contrast to the leaves of the intact plant, verbascoside, not forsythoside B, was the major phenyloethanoid glycoside found in the HRs. Its content in one of the cultivated clones reached 23.31 mg/g DW. Leucosceptoside B and martynoside were present in smaller amounts [86]. *V. nigrum* L. was transformed with the *R. rhizogenes* strain ATCC 15834 using the sonication-assisted method. By means of NMR spectroscopy, metabolites from the leaves of parent plant and metabolites synthesized by the obtained HRs were compared, revealing that 5-CQA, harpagide, and harpagoside present in the leaves are absent from the HRs [87]. Similar analysis was performed for the HRs of *V. eriophorum* Godr. and leaves of the parent plant. The iridoids harpagide, aucubin, and aucubin glycosides were found only in the leaves, whereas the HRs were characterized by much higher contents of verbascoside and martynoside [88].

The current knowledge on the biosynthesis of salidroside and attempts to achieve biotechnological production of the compound from plant tissue cultures and genetically engineered microorganisms have been recently summarized by Grech-Baran et al. [89] and Liu et al. [90]. Cell suspension culture of *Rhodiola crenulata* (Hook.f. & Thomson) H.Ohba (Crassulaceae) that accumulates 26.48 mg/g DW of salidroside and compact callus of *R. sachalinensis* Boriss. containing 41.94 mg/g DW of the compound are among the best salidroside producers [89,90]. According to the WFO (The World Flora Online), *R. sachalinensis* Boriss. should be treated as a synonym of *R. rosea* L., the most reputed species of the *Rhodiola* genus. Botanical description of the plant as well as information on its distribution, ethnobotany, and medicinal properties is easy to find in the literature [65,91,92]. In one study, the HRs of *R. sachalinensis* were obtained via the infection of leaf explants with *R. rhizogenes* strain A4. The transformed roots cultivated in a liquid ½ MS medium, accumulated 4.2 mg/g FW of salidroside. Upon the elicitation with *Aspergillus niger*, *Coriolus versicolor*, or *Ganoderma lucidum* (0.05 mg/L), the content of salidroside increased to 7.1 mg/g FW, and a better growth of the roots was observed. A similar effect (6.6 mg/g DW salidroside and better growth) was achieved with the precursor feeding (tyrosol, tyrosine, phenylalanine; 1 mM/L) [93].

From roots and in-vitro-cultured MeJa-treated cells of *R. sachalinensis*, two putative UDP-glucosyltransferase (UGT) cDNAs were isolated: *UGT72B14* and *UGT74R1*. Overexpression of the genes in the HRs of *R. sachalinensis* led to the enhanced production of salidroside. The roots overexpressing *UGT72B14* contained 19.81 mg/g DW of the phenylethanoid glycoside. The content was 420% higher than that in the control HRs transformed with an “empty” vector [94].

The HRs of *R. crenulata*, when elicited with MeJa or SA, demonstrated an increase in salidrose content and a corresponding increase in the expression levels for the genes encoding tyrosine decarboxylase (TDC) and UGT. Overexpression of *RcTDC* in the genetically engineered HRs led to the enhanced accumulation of tyramine, tyrosol and salidroside [95]. Tyrosine aminotransferase (TAT) catalyzes the conversion of tyrosine into 4-hydroxyphenylpyruvate. Suppression of *RcTAT* in genetically engineered *R. crenulata* HRs caused increased salidroside production, suggesting that the enzyme may be engaged in a competing metabolic pathway [96].

Without the precursor (tyrosol) feeding, the HRs of *R. kirilowii* (Regel) Maxim. were found not to produce salidroside. Supplementation of the nutrient medium with tyrosol triggered the activity of tyrosol glucosyltransferase and salidroside biosynthesis. Measurements of tyrosol glucosyltransferase activity in the HRs revealed the maximum activity of the enzyme on day 18 of the culture. Tyrosol (2.5 mM) added on that day was converted into salidroside at the maximum rate [97]. Supplementation of the *R. kirilowii* HRs with cinnamic acid resulted in the rosavin (cinnamyl diglycosides) biosynthesis. In the optimum conditions (addition of 2.5 of mM cinnamic acid on the inoculation day and extra 1% sucrose on the 14th day of culture) the HRs accumulated 0.7 mg/g DW of rosarin and 14.0 mg/g DW of rosavin in the biomass, and released nearly 500 mg/L of rosavin to the culture medium. Exogenously applied cinnamic acid, however, hampered the growth of the HRs [98]. Salidroside content in the HRs of *R. quadrifida* was shown to be low (0.58–0.71 mg/g DW), but the rhizomes of the intact *R. quadrifida* plant accumulated much less salidroside than did those of R. rosea plants [99].

The HRs that accumulated high contents of phenylethanoids are listed in Table 2.

### 2.3. Hydroxycinnamates

Derivatives of hydroxycinnamic acids are ubiquitous in the plant kingdom. This subclass of polyphenolics includes acylquinic acids (with the most known chlorogenic acids) and conjugates of cinnamic acid derivatives with other organic acids (e.g., tartaric, shikimic, aldaric, dihydroxyphenyllactic) and with sugars. They are the common constituents of food plants (like blueberries, apples, sweet cherries, grapes, spinach, chicory, lettuce, and some culinary herbs). Coffee, ciders, and wines are also rich dietary sources of hydroxycinnamates. The importance of this group of polyphenols for human health is a subject of extensive research [16,30,100].

#### 2.3.1. Conjugates with Tartaric Acid

**Figure 3:**
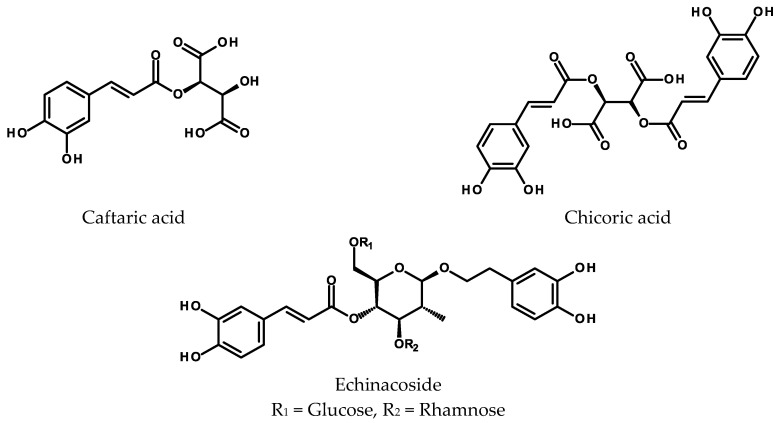
Chemical structures of caftaric acid, chicoric acid, and echinacoside.

Two compounds of this type have been described as metabolites of the HRs: caftaric (caffeoyltartaric acid, CTA) and chicoric (dicaffeoyltartaric, DCTA) acid (Figure 3). Recently, the chicoric acid biosynthetic pathway in *Echinacea purpurea* (L.) Moench (purple coneflower, Asteraceae) has been proposed by Fu and coworkers, with the HRs being one of the tools used in this research [101]. The final step in the biosynthesis takes place in the vacuole and is performed by a serine carboxypeptidase-like (SCPL) acyltransferase that uses chlorogenic acid as an acyl donor (instead of 1-O-caffeoyl-*β*-D-glucose) and transfers the caffeoyl group to CTA to form DCTA. CTA is synthesized in cytosol from caffeoyl-CoA and tartaric acid in a reaction catalyzed by an acyltransferase from the BAHD family.

The data on the pharmacological activity of CTA are sparse in comparison to those concerning DCTA. CTA counteracts oxidative damage of biological macromolecules and reduces inflammatory response. Moreover, it ameliorates indomethacin-induced gastric ulcer in rats [102,103]. DCTA enhances glucose uptake, diminishes insulin resistance and oxidative stress, demonstrates anti-inflammatory and antiviral activity, improves brain and liver functions, and may have beneficial effects in atherosclerosis and osteoarthritis [104,105,106,107]. In addition to ameliorating cognitive impairment via the promotion of the antioxidant defense system and preventing memory impairment and amyloidogenesis caused by systemic inflammation [108,109], DCTA improves neuron survival against inflammation [110].

Numerous attempts have been made to achieve the mass propagation of purple coneflower through in vitro methods and to start the biotechnological production of caffeoyltartaric acids from in vitro cultures of *E. purpurea* [111,112,113]. They have been lately summarized by Hussain et al. [5] in their comprehensive review on plant root cultures. The first HR cultures of *E. purpurea* were obtained by inoculation of hypocotyls of etiolated seedlings with *R. rhizogenes* strains ATCC 15834 and R1601. Hypocotyls infected with strains LMG63 and LMG150 produced only callus tissue. Alkamides [114] were the specialized metabolites analyzed in this study and no data on hydroxycinnamate content were provided [115]. The HRs initiated from leaf explants of purple coneflower with the *R rhizogenes* strain ATCC 43057 and cultivated in MS medium for 50 days produced up to 12.2 g/L of dry biomass, if extra sucrose was added on day 40. The roots contained DCTA (19.21 mg/g DW), CTA (3.56 mg/g DW), and 5-CQA (0.93 mg/g DW) [116]. The HRs transferred to continuous light (60 μmol/m^2^ s) did not show altered biomass accumulation but started to accumulate anthocyanins. Moreover, the contents of caffeoyltartaric acid rose markedly (DCTA up to 27 mg/g DW; CTA up to 6 mg/g DW). The increase in caffeic acid derivative content was correlated with a higher activity of PAL [117]. Addition of gibberellic acid (GA_3_; 0.025 μM) to the nutrient medium caused a further increase in PAL activity and caffeoyltartaric acid accumulation (DCTA: c. 36 mg/g DW; CTA: c. 7.5 mg/g DW). The content of 5-CQA was similar to that of CTA [118]. The roots grown in an airlift bioreactor with increased aeration (volume 17 L; 30 days growth cycle) yielded 9 g/L DW of biomass and a maximum 146.5 mg/L of DCTA (16.3 mg/g DW). An exposure to ultrasound (6 min at day 20) resulted in higher biomass accumulation (12.8 g/L) and higher DCTA content (178.2 mg/L) [119,120]. Sonication of the HRs grown in flasks (15-day-old roots sonicated for 6 min) caused a nearly 30% higher content of DCTA in the sonicated HRs compared to that in the control roots [121]. Alterations in macronutrient contents in ½ MS medium did not improve the productivity of the HRs obtained from leaf explants inoculated with the *R. rhizogenes* ATCC 15834 [122]. Demirci et al. [123] intiated the HR culture of purple coneflower from leaf explants inoculated with the *R. rhizogenes* strain ATCC 43057. The roots were maintained in the dark and subcultured every 21 days. After the initial 10 days of growth, the cultures were supplemented either with 24-epibrassinolide (0.5, 1.0, and 2.0 mg/L) or with L-phenylalanine (100, 500, and 1000 μM). DW, FW, and the metabolite contents in the roots were assessed every 10 days up to the 50th day of culture. Neither 24-epibrassinolide nor L-phenylalanine demonstrated detrimental effects on the HRs growth at the concentration range applied. The maximum DCTA content (20.2–24.1 mg/g DW) was achieved with 24-epibrassinolide treatment. Both treatments were advantageous for CTA and echinacoside production. This was the first study to describe the echinacoside production in *E. purpurea* HRs. Earlier, the compound was obtained from the adventitious root culture of *E. angustifolia* DC. [124].

Although DCTA is a major constituent of lettuces, neither the *Lactuca virosa* L. nor the *L. indica* L. (Asteraceae) HRs have been found to accumulate significant amounts of the compound [125,126].

Table 3 shows the HR cultures that accumulated high contents of CTA and DCTA.

#### 2.3.2. Conjugates with Quinic Acid

Esters of *trans*-hydroxycinnamic acids with 1L-(-)-quinic acid synthesized and accumulated by plants are known as chlorogenic acids (CGAs). In the 1970s, new rules for describing the structure of 1L-(-)-quinic acid were recommended by the IUPAC, resulting in changes in the nomenclature of the compounds. According to IUPAC rules, one of the most common caffeoylquinic acids, chlorogenic acid, should be denoted as 5-O-caffeoylquinic acid (5-CQA). Formerly, the compound was known as 3-O-caffeoylquinic acid (3-CQA). Unfortunately, there is still no consensus regarding the nomenclature [16,127,128]. In this review, the IUPAC rules have been applied, but the numbering system used by the authors of particular papers cited herein is not always clear.

**Figure 4:**
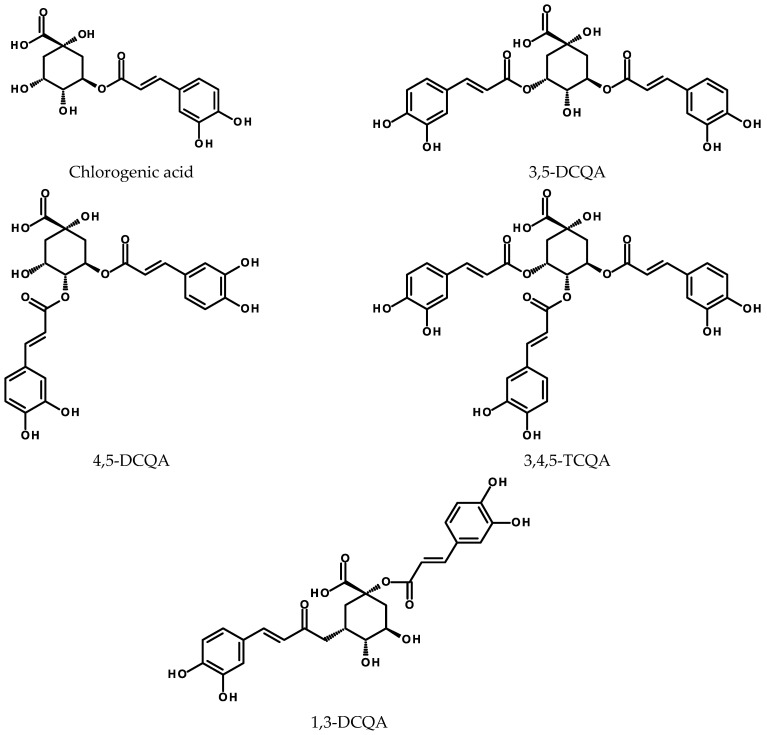
Chemical structures of selected acylquinic acids (CGAs).

CGAs (Figure 4) are common constituents of food. Their daily intake in coffee drinkers is estimated to be 0.5–1.0 g (a cup of Espresso coffee contains about 200 mg of CGAs) [129]. Other than coffee, the main food contributors of CGAs are potatoes, apples, artichokes, plums, cherries, prunes, tomatoes, and carrots [130]. Questions concerning the bioavailability of CGAs have been summarized elsewhere [16,131]. One of the most recent studies on the subject examined the metabolic fate of artichoke polyphenols (mainly caffeoylquinic acids) after ingestion by healthy volunteers [132]. CGAs are the antioxidative and anti-inflammatory agents that demonstrate neuroprotective effects, prevent hypoxia-induced retinal degeneration, and counteract the formation of advanced glycation end products [16,128,133,134,135,136,137,138].

Although some of the CGAs can be easily obtained from crop plants, attempts have been made to achieve their production in plant tissue cultures [139].

Chlorogenic acid, an easy-to-detect and widely occurring plant metabolite, has been frequently described as a constituent of the HRs. Its content, however, is usually low. Some examples of 5-CQA occurrence in the HRs are given in Section 2.1. (*Ficus carica*, *Leonotis nepetifolia*, *Leonurus sibiricus*, *Mentha spicata*, *Panax quinquefolius*). The compound is engaged in plant defense against infections and insect infestation [140,141]. Yadav et al. [142] studied the interactions between *R. tumefaciens* and phenolic compounds exuded by cotyledonary nodal explants of *Cicer arietinum* L., the species that demonstrates recalcitrance to *Rhizobium*-mediated transformation. The inoculation with *R. tumefaciens* enhanced the production of some phenolics (including 5-CQA) in plant explants and the subsequent liberation of the compounds to the culture medium that in turn inhibited the growth of the bacterial vector and adversely affected the process of genetic transformation.

In another study, CGAs were detected in the HRs of *Leontopodium alpinum* Cass. (synonym of *Leontopodium nivale* subsp. *alpinum* (Cass.) Greuter; Asteraceae); however, they were not quantified [143]. 

The HRs of *Lactuca virosa* L. (Asteraceae), obtained by the infection of leaf explants with the *R. rhizogenes* strain LBA 9402 and cultivated in a modified MS medium in the dark were found to produce 3,5-DCQA (3,5-di-O-caffeoylquinic acid) as a major phenolic metabolite (25.8 mg/g DW). The content of 3,5-DCQA was much higher than that in the intact plant organs and callus tissue. The HRs also produced 5-CQA (11.3 mg/g DW) and DCTA (7.9 mg/g DW). Leaves of the intact *L. virosa* plant contained twice as much DCTA [125]. *L. serriola* L. transformed with the *R rhizogenes* strain AR15834 developed HRs that exhibited a higher total reducing capacity than did the untransformed roots. The CGA content in the roots was not analyzed [144]. *L. indica* was transformed using the *R. rhizobacterium* strain R1000. The developed roots accumulated 3,5-DCQA, DCTA, and 5-CQA, albeit in amounts lower than those found in *L. virosa*. The best results were achieved using MS medium and 100 μM of MeJa as an elicitor. The maximum contents of the CGAs and DCTA were detected 72 h after the addition of MeJa (3,5-CQA, c. 0.14 mg/g FW; DCTA, c. 0.14 mg/g FW; 5-CQA, c. 0.02 mg/g FW) [126].

Chicory (*Cichorium intybus* L.; Asteraceae) is closely taxonomically and chemically related to lettuces. In one study, the HRs of the plant were obtained by inoculation of leaf explants with the *R. rhizogenes* strain LBA 9402 and grown in a modified MS medium either under a photoperiod (cool white fluorescent tubes, 20 μE m^−2^ s^−1^, 16 h light) or in the dark. Exposure to light was advantageous for CGA production in the HRs. At the stationary phase of growth, the roots cultivated under the photoperiod contained 55.7 mg/g DW of 3,5-DCQA, 9.4 mg/g DW of 5-CQA, and 1.3 mg/g DW of DCTA [145].

In another study, leaves of *C. intybus* var Orchies were inoculated with the *R. rhizogenes* strain 2659 to obtain the HRs. The roots were grown in ½ MS medium under a 16 h light photoperiod. After the elicitation with MeJa (on day 12), production of CGAs was strongly promoted, and 12 days after the addition of MeJa (0.15 mM), the content of 3,5-DCQA in the HRs reached 12% DW (120 mg/g DW). From the elicited roots, 3,4,5-tri-O-caffeoylquinic acid was isolated, the compound formerly undetected in the HRs of chicory. Unelicited roots produced approximately 10 mg/g DW of 5-CQA and 50 mg/g DW of 3,5-DCQA [146].

*Eclipta prostrata* (L.) L. (Asteraceae) HRs, in other research, were obtained via the infection of aseptic seedlings with *R. rhizogenes* LBA 9796 and were elicited with jasmonic acid (JA) and MeJa to enhance production of wedelolactones and 3,5-DCQA. The roots, after 4 days of growth in the presence of either 100 μM of JA or 140 μM of MeJa, accumulated 44.71 mg/g DW and 41.62 mg/g DW of 3,5-DCQA, respectively. Control cultures contained 18.08 mg/g DW of the compound [147]. Lopes et al. [148] investigated the biosynthesis of demethylwedelolactone, wedelolactone, and 3,5-DCQA in the HRs of *E. prostrata*. Feeding with [3-^13^C]_DL_-phenylalanine proved that in contrast to wedelolactones, 3,5-DCQA molecule originates entirely from the shikimate pathway.

Young, aseptic leaves of *Stevia rebaudiana* Bertoni (Asteraceae) var. FengNong 3 were inoculated with *R. rhizogenes* strain C58C1 in one study. The developed HRs were cultivated in ½ MS medium under a 12/12 h (light/dark) photoperiod. The root clone that showed the best growth yielded 15.23 g/100 mL of FW and 1.54 g/100 mL DW of biomass, after 24 days of culture. Roots of the clone accumulated chlorogenic acid (39.41 mg/g DW) and 3,5-DCQA (48.10 mg/g DW). Some of the obtained root clones produced up to 4.29 mg/g of 4,5-DCQA. The optimum for both CGA production and root growth was the B5 medium [149].

A total of 11 CGAs produced by the HRs of *Rhaponticum carthamoides* (Willd.) Iljin (Asteraceae) transformed with *R. rhizogenes* strain A4 were tentatively identified using UPLC-PDA-ESI-MS^3^ analysis. 5-CQA (5.12 mg/g DW), 3,5-DCQA (3.08 mg/g DW), and one of the two tri-O-caffeoylquinic acid derivatives (5.97 mg/g DW) were the most abundant CGAs from the roots cultivated in the presence of light. The HRs cultivated in the dark produced less CGAs [150]. An extract from the HRs effectively induced apoptosis in human glioma primary cells [151].

CGAs produced by the HRs of *Polyscias filicifolia* L.H.Bailey (Araliaceae) transformed with *R. rhizogenes* strain ATCC 15834 were tentatively identified using the HPLC-PDA-ESI-MS method and nine *p*-coumaroylquinic, caffeoylquinic, and feruloylquinic acids were found in the culture. The HRs elicited for 1 day with 100 μM of MeJA and then transferred to control medium for 30-day cultivation produced up to 1.6 mg/g DW of chlorogenic acid [152].

The *Hypericum perforatum* L. (Hypericaceae) HRs transformed with *R. rhizogenes* strain A4M70GUS were found to accumulate the following monoacylquinic acids: chlorogenic acid (0–6.94 mg/g DW), *p*-coumaroylquinic acid (0.04–0.09 mg/g DW), and feruloylquinic acid (0.11–0.94 mg/g DW). The contents of CGAs depended on the analyzed root line and were much lower than those in the shoots regenerated from the HRs [153].

The HRs that accumulated high contents of CGAs are shown in Table 4.

#### 2.3.3. Rosmarinic Acid, Salvianolic Acids, and Miscellaneous Compounds

**Figure 5:**
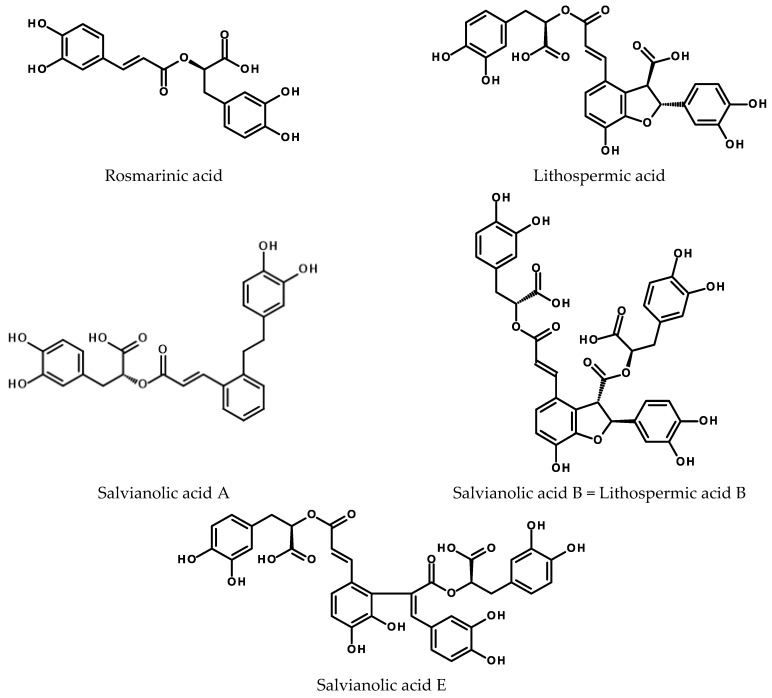
Chemical structures of rosmarinic acid and selected salvianolic acids.

Plant polyphenolics of this group are typically associated with Lamiaceae plants. The seeds, leaves, and roots of many species included in the family are widely used either as spices (mint, basil, savory, rosemary, oregano, marjoram) or as medicines (lemon balm, lavender, thyme). Rosmarinic acid (RA) and salvianolic acids (see Figure 5), the compounds of potential use in medicine and cosmetics, have attracted much attention. Their biosynthesis, distribution in the plant kingdom, biotechnological production, and activity have been the subject of several review papers [154,155,156,157,158].

*Ocimum basilicum* L. (Lamiaceae), a medicinal and aromatic plant commonly used as a spice, is a source of RA and other valuable metabolites. Studies on the genus *Ocimum* have been recently summarized by Pandey et al. [159]. Research on HRs of *Ocimum* spp. began in the 1990s.

In one study, two agropine and three mikimopine clones of the HRs were obtained by inoculation of leaf discs from *Ocimum basilicum* L. with two different strains of *R. rhizogenes*: ATCC 15834 and MAFF 03-01724. The root clones were cultured in the dark in three different nutrient media: MS, B5, and WP (woody plant medium). The highest DW (450–700 mg), after 6-week culture, was achieved with the MS medium. The roots, depending on the clone, contained 2.5–7.0% DW of RA, except for one highly productive clone (c. 12% DW RA). The HRs grown in WP and B5 medium provided c. 400 mg DW of biomass at the end of the culture cycle. The RA content in the roots was 0–14% (B5 medium) and 6–12% (WP medium). Lithospermic acid (up to 1.5% DW) and lithospermic acid B (0–0.75% DW) were also found in the culture [160]. Bais et al. [161] infected axenic plantlets of *O. basilicum* with the *R. rhizogenes* strain ATCC 15834 to start the HR cultures of the plant. The established culture, grown in MS medium and subcultured every 4 weeks, produced 3% FW of RA. The elicitation attempts with SA (50, 100 and 200 μM), MeJa (100, 200 and 500 μM), and chitosan (0.10, 0.12, and 0.15%), added at the beginning of culture cycle, failed due to the detrimental effect of the elicitors on both the growth of roots and RA production. The HRs elicited with cell wall extracts from *Phytophthora cinnamoni* and *P. drechsleri* (1, 2, and 3%) exhibited enhanced accumulation of biomass (up to 105 g/L FW) and increased RA production (up to 8.1% FW). RA was not liberated to the culture medium. Moreover, antifungal activity of RA (50–250 μM) was proven against *Pythium ultimum*, *Phytophthora aphanidermatum*, *Aspergillus niger*, and *Fusarium oxysporum*. Four strains of *R. rhizogenes* were used to obtain HRs from three cultivars of *O. basilicum* (Subja, Holy Green, and Red Rubin). The highest biomass of roots (114-116 mg DW from one explant) was achieved after 60 days of growth, but the maximum RA content (66.2–71.0 mg/g DW) was found 40 days after the inoculation of the HRs into the fresh medium [162]. Coculture of the HRs with arbuscular mycorrhizal fungus *Rhizophagus irregularis* (earlier *Glomus intraradices*) resulted in a significantly higher biomass and RA accumulation [163]. Kwon et al. [164] investigated the HRs of purple and green basil. They found that although light favors both biomass and RA production in green basil, the purple cultivar used in the study demonstrated better productivity when grown in the dark. The cultures of red basil (*O. basilicum* cv. “Purpurascens’) accumulated less RA than did those of the green cultivar (*O. basilicum* cv. “Cinnamon”).

RA and nine phenolic acids were identified as constituents of the *Hyssopus officinalis* L. (Lamiaceae) HRs obtained by inoculation of leaf explants with *R. rhizogenes* LBA 9402 [160a]. The highest RA content, 6% DW (c. 0.9 g/L), was detected in the HRs grown in GB5 medium containing 10% sucrose [165]. 

The *Dracocephalum kotschyi* Boiss (Lamiaceae) HRs were induced by infecting with *R. rhizogenes* LBA 9402 in one study. The roots synthesized a number of polymethoxylated flavonoids and RA (up to 1.4 mg/g DW) [166]. Internodal explants of *D. forrestii* W.W. Smith, infected with the *R. rhizogenes* strain A4, developed HRs that produced up to 20 mg/g DW of RA and up to 6.9 mg/g DW of salvianolic acid B. The optimum for the polyphenols production was a WP medium containing 3% sucrose. The HRs were grown in the dark [167]. The maximum RA content (1.2 mg/g FW) in the HRs of *D. kotschyi* was achieved via elicitation with Fe_3_O_4_ nanoparticles (75 mg/L, 24 h) [168].

From the HRs of *Nepeta teyeda* Webb et Berth. (Lamiaceae) transformed with *R. rhizogenes* ATCC 15834, formerly unknown compounds, including the sesquiterpene (-)-cinalbicol, the diterpene teydeadione, a degraded C23-triterpene (teydealdehyde), and three fatty acid esters of lanosta-7,24-dien-3*β*-ol, were isolated together with two dehydroabietane diterpenes, eight triterpenes, and eighteen known phenolic compounds. Moreover, the propyl ester of rosmarinic acid was isolated for the first time from a natural source [169]. The HRs of *N. cataria* L. produced up to 13.6 mg/g DW of RA. Indole-3-butyric acid (IBA) added to the culture medium (0.5 mg/L) increased accumulation of RA in the roots (19.2 mg/g DW). A similar result was achieved via treatment with putrescine (50 mg/L) [170,171].

Undifferentiated cultures of *Coleus* spp and *Plectranthus* spp. (Lamiaceae) have been shown to be excellent producers of RA [154,172]. The *C. forskohlii Briq*. HRs, induced by *R. rhizogenes* MAFF 03-01724, accumulated up to 9.5 mg of RA per flask (c. 4g FW) one week after elicitation with 100 μM of Meja [173]. *C. blumei* Benth. (*Plectranthus scutellarioides* R.Br.) infected with *R. rhizogenes* A4 developed HRs that produced c. 50 mg/g DW of RA. Upon elicitation with MeJa (100 μM, 8 days), the RA content rose to c. 80 mg/g DW [174]. A synthetic gene encoding *β*-cryptogein (a peptide implicated in a response to pathogens) was introduced into the HRs of *C. blumei* with a *R. rhizogenes* A4-carrying binary vector. The roots with *β*-cryptogein expression liberated phenolics to the culture medium. The amount of liberated RA reached 0.25 mg per 1 L of the medium [175]. Effects of hydroxyphenylpyruvate reductase (HPPR) and rosmarinic acid synthase (RAS) overexpression and suppression were studied in *C. blumei* HRs transformed with *R. rhizogenes* ATCC 15834 carrying different genetic constructs. The *HPPR*-overexpressing line with increased HPPR *m*RNA levels accumulated more RA (3% DW) than did the control roots (c. 1.8%) [176].

The *Prunella vulgaris* L. (Lamiaceae) HRs transformed with *R. rhizogenes* ATCC 15834 were found to accumulate 15–30 times more RA than did the original *P. vulgaris* plant. The content of RA in the HRs (c. 40 mg/g DW) increased up to c. 60 mg/g DW when the roots were treated with either 0.2 mg/L of ethephon for 5 days or with 6.9 mg/L of SA for 2 days. The relative expression levels of the genes encoding PAL and HPPR, 2–8 days after exposure of the HRs to the elicitor, were significantly higher than were those in the control roots [177]. Tyrosine aminotransferase engaged in the RA biosynthesis in *P. vulgaris* (PvTAT) was isolated and described from root of the plant. Antisense- and sense *PvTAT*-expressing hairy root lines were developed, and *PvTAT* gene expression levels, TAT enzyme activity, and RA content were analyzed in the obtained root clones. Antisense-expressing HRs demonstrated a reduced TAT activity and a decrease in RA content. Overexpression of *PvTAT* led to the enhanced accumulation of RA (up to 70 mg/g DW) [178]. The HRs of *P. vulgaris* transformed with ATCC 15834 contained over 80 mg/g DW of RA (control roots c. 50 mg/g) after the elicitation with 100 μM of MeJa (5 days) [179].

Leaf and stem explants of *Agastache rugosa* Kuntze (Lamiaceae), after inoculation with *R. rhizogenes* R1000, were reported to develop HRs that produced 14.1 g DW/L of biomass and 116 mg/g DW of RA after two weeks of culture in MS medium containing 3% sucrose. The roots were maintained in a growth chamber with a 16 h photoperiod [180]. *Lepechinia caulescens* (Ortega) Epling (Lamiaceae) roots transformed with *R. rhizogenes* ATCC 15834 in optimum conditions (MS salts, B5 vitamins, 4.5% sucrose) produced 41.7 mg/g DW of RA. The content increased to c. 60 mg/g DW after the elicitation with 200 μM of MeJa (24 h) [181]. Numerous HRs of the Lamiaceae plants were established for the active metabolite production, including the formerly described HRs of *Mentha spicata* (70 μg/g DW of RA and 60 μg/g DW of lithospermic acid B) [57] and *Leonotis nepetifolia* (2.6 mg/g DW of RA) [58].

It is worth mentioning the biosynthesis of RA and lithospermic acid B (synonyms: monardic acid B and salvianolic acid B) in the HRs of *Rindera graeca* (A.DC.) Boiss. & Heldr. (Boraginaceae). It was found that the HRs contained up to 33.7 mg/g of RA and up to 106.1 mg/g DW of lithospermic acid B on the 30th day of the culture cycle. Cold stress increased concentration of the polyphenolics in the roots [182].

Numerous papers have reported on the HRs of different *Salvia* species: *S. bulleyana* Diels, *S. nemorosa* L., *S. officinalis* L., *S. przewalskii* Maxim., *S. virgata* Jacq., *S. viridis* L., and the most popular, *S. miltiorrhiza* Bunge, a renowned remedy of TCM. Several dozen of papers on the engineering of the secondary metabolism in *S. miltiorrhiza* have been published since 2015, and the volume of data clearly deserves separate review. This is why we have decided to exclude this species from the current paper. 

The HRs of *S. officinalis*, obtained by infection of sterile shoots of the plant with *R. rhizogenes* ATCC 15834, were found to contain up to 45 mg/g DW of RA. The sage HRs that were cultivated in the dark tended to accumulate less RA than did the roots maintained under a photoperiod (16 h light) [183]. Methanolic extract from the HRs of *S. officinalis* contained 30.9 mg/g DW of RA and demonstrated high antioxidative and radical scavenging activity and in some assays, higher than those of roots and shoots of the in-vivo-grown plants [184]. After the transfer to laboratory-scale sprinkle nutrient bioreactor, the sage HRs produced 34.7 mg/g DW (477.1 mg/L) of RA [185]. The HRs of *S. nemorosa*, cultivated in ½ MS medium containing 3% sucrose accumulated over 50% less RA (15.9 mg/g DW) [186]. Ag^+^ ions, YE, and MeJa were applied to enhance the productivity of the *S. virgata* HRs. The best results were achieved with MeJa (72 h) at a final concentration of 22.4 ppm. The elicited roots produced up to 18.5 mg/g DW of RA and up to 2.1 mg/g DW of salvianolic acid A [187]. MeJa at a final concentration of 400 μM and SA (50 μM) were applied to stimulate the growth and production of polyphenolics in the HRs of *S. przewalskii.* The roots treated with MeJa, 3 days after addition of the elicitor, contained 67.1 mg/g DW of RA and 21.4 mg/g DW of salvianolic acid B. Moreover, the elicited roots produced increased amounts of tanshinones (diterpenoids). The accumulation of polyphenolics in the elicited HRs of *S. przewalskii* was accompanied by the elevated transcription levels of *PAL*, *4CL*, *TAT*, *HPPR*, and *RAS* [188]. The HRs of *S. viridis* accumulated up to 35 mg/g DW of RA and minor quantities of other polyphenolics, tentatively identified by UPLC-PDA-ESI–MS^2^ as prolithospermic acid, salvianolic acid J, two hexosides of RA, salvianolic acid E, methyl rosmarinate, and two isomers of salvianolic acid F [189]. An attempt to optimize the culture conditions with the proper choice of a nutrient medium formulation and the appropriate light regime was also made [190]. Leaves and shoots of the axenic *S. bulleyana* plants were infected with *R. rhizogenes* A4 to obtain the HRs. The growth index of the HRs, measured after five weeks of growth in a liquid WP medium, was 8–14, depending on the clone. The RA content in the HRs reached 40 mg/g DW, and this compound dominated the polyphenolic fraction in the roots. Minor quantities of salvianolic acids E, K, and F and methyl rosmarinate were also detected [191]. The roots were elicited with MeJa, YE, *trans*-anethole, and CdCl_2_. Chromium salt had a detrimental effect on both biomass and polyphenol accumulation. The most effective method for the induction of the enhanced RA production (over 100 mg/g DW) was the application of MeJa (100 μM, 3 days). In the elicited roots, the increased accumulation of caffeic acid preceded the increased production of polyphenolics [192]. Optimization of the culture conditions for the *S. bulleyana* HRs revealed the advantageous effects of diminished sucrose concentration (2%), diminished contents of salts and vitamins, and the lack of illumination on polyphenol accumulation in the biomass. In the optimum conditions, the roots produced over 90 mg/g DW of RA and over 10 mg/g DW of salvianolic acid K [193].

The HR cultures that produced high yields of RA, lithospermic acid B, and salvianolic acid K are listed in Table 5.

## 3. Conclusions

Table 1, Table 2, Table 3, Table 4 and Table 5 summarize the most remarkable achievements of the research on highly productive hairy root cultures containing phenolic/polyphenolic antioxidants from the simple phenolics, phenylethanoid, and hydroxycinnamate group. Rosmarinic acid production by the HRs of the Lamiaceae plants has been shown to be one of the best examples of the biosynthetic potential of the HRs. The high yields of the phenolic/polyphenolic natural products in the HRs were obtained using procedures such as the optimization of culture conditions, selection of the optimum bacterial strain and plant material to initiate the HRs, elicitation, supplementation with phytohormones, precursor (feeding, and genetic engineering (overexpression, heterologous expression, or silencing of the genes encoding the regulatory transcription factors and biosynthetic enzymes). In the analyzed experimental works, the elicitation with methyl jasmonate was the most extensively used method for stimulation of polyphenolics production. Cell wall preparations from fungal or bacterial pathogens also showed some potential. However, the high contents of polyphenols in the HRs may be accompanied by poor root growth. This may be unfavorable for the potential applications of the cultures that accumulate products in the biomass.

The HR cultures proved to be a valuable tool for the characterization of regulatory mechanisms in plant phenol/polyphenol biosynthesis. They were used for elucidation of the functions of particular biosynthetic enzymes and transcription factors. At present, the HR cultures are primarily useful in the genetic engineering of plants. Despite the efforts to maximize production of valuable phenolics/polyphenolics using the HRs, the implementation of the industrial processes using HRs to produce antioxidant natural products seems to be limited not only by economic issues but also by the current regulations concerning genetically transformed cultures.

## Figures and Tables

**Table 1:** Hairy root cultures that produced high yields of simple phenolics and phenolic acids.

Plant Species	Hairy Roots	Natural Product	Maximum Content in the Biomass	Literature
*Datura stramonium* L.	Expressing *pmHCHL*	*p*-Hydroxybenzoic acid *O*-*β*-D-glucoside *p*-Hydroxybenzyl alcohol *O*-*β*-D-glucoside	4.4 mg/g FW 2.8 mg/g FW	[32]
*Beta vulgaris* L.	Expressing *pmHCHL*	*p*-Hydroxybenzoic acid (4-Hydroxybenzoic acid) glucose ester	140 mg/g DW	[33]
*Momordica charantia* L.	Wild type	Gentisic acid Salicylic acid	5.5 mg/g DW 2.5 mg/g DW	[45]
*Polygonum multiflorum* Thunb.	Wild type	Pyrogallol	1.4 mg/g DW	[48]
*Ficus carica* L. cv. Sabz	Wild type	Gallic acid Coumaric acid	7.5 mg/g DW 0.9 mg/g DW	[52]
*Leonurus sibirica* L.	Expressing *AtPAP1*	Chlorogenic acid Caffeic acid	19.4 mg/g DW 11.4 mg/g DW	[56]
*Leonotis nepetifolia* (L.) R. Br.	Wild type	*p*-Coumaric acid *m*-Coumaric acid	4.9 mg/g DW 2.1 mg/g DW	[58]

**Table 2:** Hairy root cultures that produced high yields of phenylethanoids.

Plant Species	Hairy Roots	Natural Product	Maximum Content in the Biomass	Literature
*Paulownia tomentosa* Steud.	Wild type	Verbascoside	94.9 mg/g DW	[73]
*Plantago lanceolata* L.	Wild type	Plantamajoside	30–80 mg/g DW	[75]
*Verbascum xanthophoeniceum* Griseb.	Wild type	Verbascoside	23.3 mg/g DW	[86]
*Rhodiola sachalinensis* Boriss.	Overexpressing *UGT72B14*	Salidroside	19.8 mg/g DW	[94]

**Table 3:** Hairy root cultures that produced high yields of caffeoyltartaric acids and echinacoside.

Plant Species	Hairy Roots	Natural Product	Maximum Content in the Biomass	Literature
*Echinacea purpurea* (L.) Moench	Wild type	Chicoric acid	27 mg/g DW	[117]
*Echinacea purpurea* (L.) Moench	Wild type, supplemented with 0.025 μM GA_3_	Chicoric acid Caftaric acid	36 mg/g DW 7.5 mg/g DW	[118]
*Echinacea purpurea* (L.) Moench	Wild type, supplemented with 1 mg/L of 24-epibrassinolide	Chicoric acid Caftaric acid Echinacoside	24.1 mg/g DW 6.9 mg/g DW 4.3 mg/g DW	[124]
*Echinacea purpurea* (L.) Moench	Wild type, supplemented with 500 μM L-phenylalanine	Chicoric acid Caftaric acid Echinacoside	17.4 mg/g DW 6.3 mg/g DW 5.4 mg/g DW	[124]

**Table 4:** Hairy root cultures that produced high yields of acylquinic acids.

Plant Species	Hairy Roots	Natural Product	Maximum Content in the Biomass	Literature
*Lactuca virosa* L.	Wild type	3,5-di-*O*-caffeoylquinic acid	25.8 mg/g DW	[125]
*Cichorium intybus* L.	Wild type	3,5-di-*O*-caffeoylquinic acid 5-*O*-caffeoylquinic acid	55.7 mg/g DW 9.4 mg/g DW	[145]
*Cichorium intybus* L. var Orchies	Wild type, elicited with 150 μM MeJa	3,5-di-*O*-caffeoylquinic acid	120 mg/g DW	[146]
*Eclipta prostrata* (L.) L.	Wild type, elicited with 100 μM of jasmonic acid	3,5-di-*O*-caffeoylquinic acid	44.7 mg/g DW	[147]
*Eclipta prostrata* (L.) L.	Wild type, elicited with 140 μM MeJa	3,5-di-*O*-caffeoylquinic acid	41.6 mg/g DW	[147]
*Stevia rebaudiana* Bertoni var. FengNong 3	Wild type	5-*O*-caffeoylquinic acid 3,5-di-*O*-caffeoylquinic acid	39.4 mg/g DW 48.1 mg/g DW	[149]

**Table 5:** Hairy root cultures that produced high yields of polyphenolic antioxidants (RA, lithospermic acid B, and salvianolic acid K).

Plant Species	Hairy Roots	Natural Product	Maximum Content in the Biomass	Literature
*Ocimum basilicum* L.	Wild type	Rosmarinic acid	120 mg/g DW	[158]
*Ocimum basilicum* L.	Wild type, elicited with 2% of *Phytophthora cinnamoni* cell wall extract	Rosmarinic acid	81 mg/g FW	[159]
*Ocimum basilicum* L.	Wild type, cocultivated with *Rhizophagus irregularis*	Rosmarinic acid	140.5 mg/g DW	[161]
*Agastache rugosa* Kuntze	Wild type	Rosmarinic acid	116 mg/g DW	[178]
*Rindera graeca* (A.DC.) Boiss. & Heldr.	Wild type	Lithospermic acid B	106.1 mg/g DW	[180]
*Salvia przewalskii* Maxim.	Wild type	Rosmarinic acid Lithospermic acid B	67.1 mg/g DW 21.4 mg/g DW	[186]
*Salvia bulleyana* Diels	Wild type	Rosmarinic acid	110.2 mg/g DW	[190]
*Salvia bulleyana* Diels	Wild type	Rosmarinic acid Salvianolic acid K	90 mg/g DW 10 mg/g DW	[191]

## Data Availability

Not applicable.

## Abbreviations

3:5-DCQA	3,5-Dicaffeoylquinic acid
4CL	4-Coumarate:CoA ligase
5-CQA	5-Caffeoylquinic acid
AChE	Acetylcholinesterase
ALDH	Alcohol dehydrogenase
AOX	Alternative oxidase
BChE	Butyrylcholinesterase
C3H	Coumaroylquinate (coumaroylshikimate) 3’-monooxygenase
C4H	Cinnamate-4-hydroxylase
CGAs	Chlorogenic acids
CTA	Caftaric acid
CuAO	Copper-containing amine oxidase
DCTA	Chicoric acid
DW	Dry weight
DXR	1-Deoxy-D-xylulose 5-phosphate reductoisomerase
FW	Fresh weight
GB5	Gamborg’s B5
HBD	Hydroxybenzoate dehydrogenase
HBS	*p*-Hydroxybenzaldehyde synthase
HCHL	4-Hydroxycinnamoyl-CoA hydratase/lyase
HCT	Shikimate O-hydroxycinnamoyltransferase
HPLC-PDA-ESI-MS	High performance liquid chromatography with photodiode detector electrospray ion source and mass spectrometry
HPPR	Hydroxyphenylpyruvate reductase
HR	Hairy root
MeJa	Methyl jasmonate
MS	Murashige and Skoog
PAL	Phenylalanine ammonia lyase
PAP1	Production of anthocyanin pigment 1
PG	Propyl gallate
*p*-HBA	*p*-Hydroxybenzoic acid (4-hydroxybenzoic acid)
PK	Pyruvate kinase
PPO	Polyphenol oxidase
RA	Rosmarinic acid
RAS	Rosmarinic acid synthase
SA	Salicylic acid
SHAM	Salicylhydroxamic acid
SKHD	Shikimate dehydrogenase
SOD	Superoxide dismutase
TAT	Tyrosine aminotransferase
TCM	Traditional Chinese medicine
TDC	Tyrosine decarboxylase
TyDC	Tyrosine/DOPA decarboxylase
UGT	UDP-Glucose glucosyltransferase
WP	Woody plant
YE	Yeast extract
